# Systemic Induction of the Defensin and Phytoalexin Pisatin Pathways in Pea (*Pisum sativum*) against *Aphanomyces euteiches* by Acetylated and Nonacetylated Oligogalacturonides

**DOI:** 10.3390/molecules22061017

**Published:** 2017-06-19

**Authors:** Sameh Selim, Jean Sanssené, Stéphanie Rossard, Josiane Courtois

**Affiliations:** 1 HydrISE, UniLaSalle, Beauvais, SFR Condorcet 3417, 19 Rue Pierre Waguet, BP 30313, F-60026 Beauvais CEDEX, France; 2 Current address: JS Consulting, 17c Avenue Jean Jaurès, 31290 Villefranche de Lauragais, France; j.sanssene@js-consult.fr; 3 Current address: University of Technology of Compiègne (UTC), Centre Pierre Guillaumat, Rue du Docteur Schweitzer, F-60203 Compiègne CEDEX, France; stephanie.rossard@utc.fr; 4 Laboratoire des Polysaccharides Microbiens et Végétaux, Université de Picardie Jules Verne, Avenue des Facultés, Le Bailly, F-80025 Amiens CEDEX, France; josiane.courtois@u-picardie.fr

**Keywords:** pea root rot, *Aphanomyces euteiches*, oligogalacturonides, real-time qPCR, gene expression, pea defense pathways, defensins, pisatins, phytoalexins

## Abstract

Oligogalacturonides (OGs) are known for their powerful ability to stimulate the plant immune system but little is known about their mode of action in pea (*Pisum sativum*). In the present study, we investigated the elicitor activity of two fractions of OGs, with polymerization degrees (DPs) of 2–25, in pea against *Aphanomyces euteiches*. One fraction was nonacetylated (OGs − Ac) whereas the second one was 30% acetylated (OGs + Ac). OGs were applied by injecting the upper two rachises of the plants at three- and/or four-weeks-old. Five-week-old roots were inoculated with 10^5^ zoospores of *A. euteiches*. The root infection level was determined at 7, 10 and 14 days after inoculation using the quantitative real-time polymerase chain reaction (qPCR). Results showed significant root infection reductions namely 58, 45 and 48% in the plants treated with 80 µg OGs + Ac and 59, 56 and 65% with 200 µg of OGs − Ac. Gene expression results showed the upregulation of genes involved in the antifungal defensins, lignans and the phytoalexin pisatin pathways and a priming effect in the basal defense, SA and ROS gene markers as a response to OGs. The reduction of the efficient dose in OGs + Ac is suggesting that acetylation is necessary for some specific responses. Our work provides the first evidence for the potential of OGs in the defense induction in pea against *Aphanomyces* root rot.

## 1. Introduction

Plants need to be able to recognize pathogen attacks in a timely manner in order to activate their defenses that provide protection against the infection process. The plant cell wall is the site of initial interaction with microbial pathogens. Pectin is one of the most accessible components of the cell wall and, therefore, is among the first structures to be altered upon pathogen invasion. The oligogalacturonides (OGs) are produced upon partial degradation of the pectin homogalacturon by pathogen pectinases and polygalacturonases [1]. However, OGs have been indicated as damage-associated molecular patterns (DAMPs) which may trigger plant defenses against pathogens. OGs endogenous elicitors consist of linear chains of α-(1-4)-linked d-galacturonic acid [2,3] and those with high biological activity have often a degree of polymerization (DP) between 10 and 15 [4]. This size is optimal for the formation of Ca^2+^ mediated inter molecular cross links resulting in structures called “egg boxes” that are thought to be necessary for OGs activity [5,6]. The short OGs with a DP of 2–6 have been reported in few cases to exhibit elicitor activity in tomato [7,8]; however, they appear to suppress defense responses in wheat [9]. It has been shown that the degree of OGs methylation clearly influences plant defense responses [10,11,12]. In wild strawberry (*Fragaria vesca*), partial demethylation of OGs in transgenic fruit enhanced resistance to *Botrytis cinerea* [13]. Furthermore, in wheat, Wietholter et al. [14] found a significant difference in the methyl ester distribution in OGs from cultivars susceptible or resistant to stem rust. Recently, we reported more than 57% protection in wheat against *Blumeria graminis* f. sp. *tritici* using 30% chemically acetylated or nonacetylated citrus OGs with DPs of 2–25 [15]. We found that only the acetylated OGs led to an increase in papilla-associated fluorescence and a reduction in the fungal haustoria formation, suggesting that acetylation is necessary for some specific responses. However, OGs elicit in several plant species [16,17,18] a wide range of defense responses, including induction of polygalacturonase-inhibiting protein (PGIP) [19,20], accumulation of phytoalexins [21], glucanase, and chitinase [22,23], deposition of callose, production of reactive oxygen species [15], and nitric oxide [24]. Root rot caused by the oomycete *Aphanomyces euteiches* is the major destructive soil-borne fungal disease of pea (*Pisum sativum*) with up to 80% yield loss per year. It is widespread in North America, Europe, Japan, Australia and New Zealand [25]. Oospores released from infected roots into the rhizosphere constitute the primary source of inoculum. *A. euteiches* invades the root system leading to a complete arrest of root growth and ultimately plant death. To date, disease control measures are limited to crop rotation and no resistant pea lines are available. The fact that oospores are able to remain dormant in the soil for up to 10 years reduces the effectiveness of crop rotation in decreasing the propagation of this pathogen [26]. Moreover, oomycetes are distantly related to true fungi and their particular physiology makes them insensitive to most fungicides [27]. Therefore, the development of alternative control methods against oomycetes is becoming urgent. In the present work, the efficiency of two distinct biochemical fractions of OGs to protect pea against *A. euteiches* was studied. These fractions consisted of OGs with DPs ranging from 2 to 25 (OGs − Ac) and one fraction that was chemically 30% acetylated (OGs + Ac). The systemic defense mechanisms elicited in pea roots as a response to rachis injections with OGs are discussed.

## 2. Results

### 2.1. Elicitor Effect of OG Compounds

**Figure 1:**
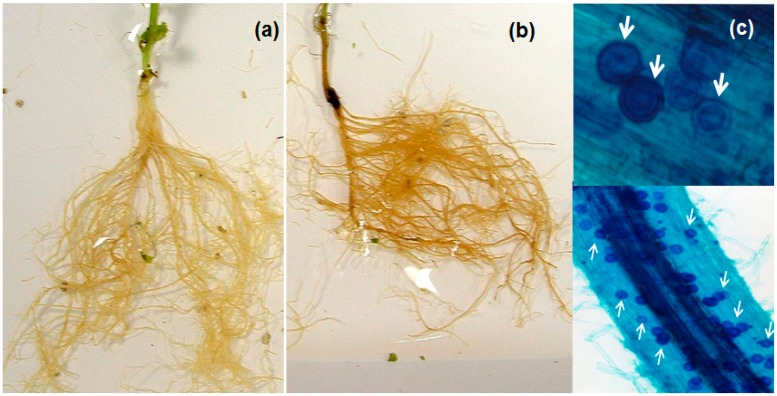
Pea stem basal part and root necrosis at 10 days after inoculation with 10^5^ zoospores of *Aphanomyces euteiches* at 5-weeks-old; (**a**) Roots of plant injected with a solution of nonacetylated oligogalacturonides (OGs − Ac) elicitor in the upper two rachises (20 µg/rachis = 40 µg/plant) at two weeks before inoculation; (**b**) Controls injected with water; (**c**) Plant roots colored with lactophenol cotton blue. *A. euteiches* oospores indicated with white arrows.

*A. euteiches* oospores were observed within necrotic tissues between 7 and 14 days after inoculation (dai). However, at ten dai, high *P. sativum*-*A. euteiches* compatibility was observed with the disease severity index (DSI) 3.5 and 76% of root fragments with more than 50 oospores (Figure 1).

**Figure 2:**
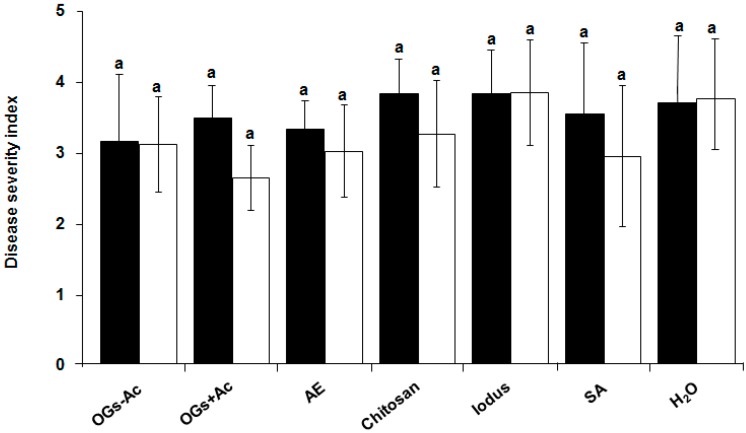
Pea root rot disease severity index at 10 days after inoculation with *Aphanomyces euteiches* at 10^5^ zoospores/plant at 5-week-old. The upper plant rachis was injected with elicitors (20 µg/rachis) at two (white) or one (black) weeks before inoculation. OGs − Ac; nonacetylated oligogalacturonides, OGs + Ac; acetylated oligogalacturonides, AE; an inoculum of *A. euteiches* zoospores (10^5^ zoospores. mL^−^^1^) heated at 100 °C for 10 min, Chitosan, Iodus, SA; salicylic acid. Controls were injected with water. The values shown are means with SD (*n* = 5). Different lower-case letters indicate significant differences between treatments according to the Tukey test (*p* ≤ 0.05).

**Figure 3:**
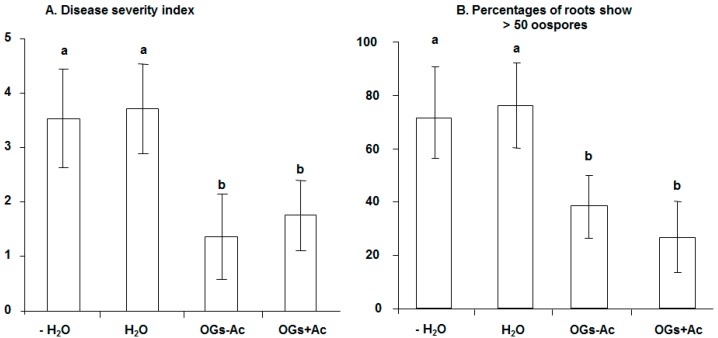
(**A**) Pea root rot disease severity index; (**B**) Percentage of roots showing >50% of oospores, at 10 days after inoculation with *Aphanomyces euteiches* at 10^5^ zoospores/plant at 5-weeks-old. The upper two rachises of plants were injected with 20 µg/rachis (=40 µg/plant) of acetylated oligogalacturonides (OGs + Ac) or nonacetylated OGs (OGs − Ac) two weeks before inoculation. H_2_O; controls injected with water, −H_2_O; controls without water injection. The values shown are means with SD (*n* = 5). Different lower-case letters indicate significant differences between treatments according to the Tukey test (*p* ≤ 0.05).

No significant difference was observed between controls injected with water at one and/or two weeks before inoculation (wbi). At 10 dai, OGs at the dose of 20 µg/plant and all other tested elicitors did not lead to any significant protection compared to the control injected with water (Figure 2). The protection level conferred on pea against *A. euteiches* was significant and increased to 43.5% and 47.8% as a response to the increase in the injected dose of OGs − Ac and OGs + Ac, respectively, to 40 µg/plant at two wbi (Figure 3a). This protection level was associated with a significant reduction (50.7% and 60%, respectively) in the percentages of root fragments containing more than 50 oospores (Figure 3b).

**Figure 4:**
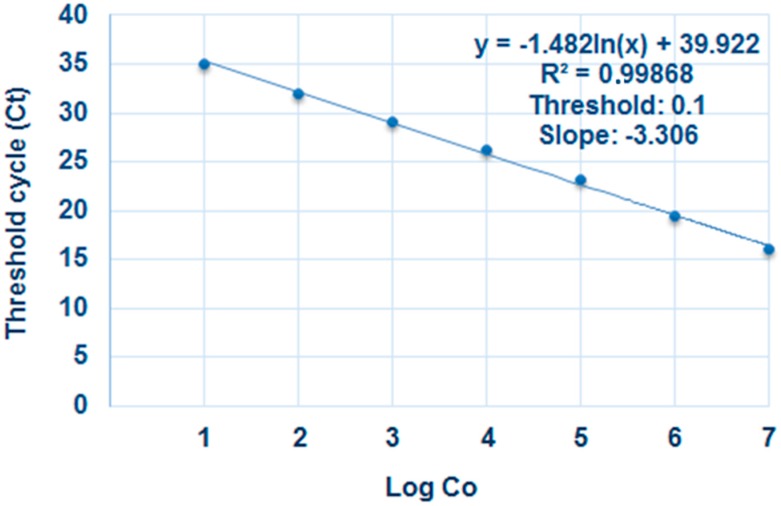
Standard curve using known copies (10^1^ to 10^7^) of the appropriate cloned target sequence of the *Aphanomyces euteiches* specific gene (GenBank accession No. AF228037.1). For each reaction, the cycle threshold (Ct), the initial cycle number at which an increase in fluorescence above a baseline can be detected, is plotted against the log10 (Log Co) of the *A. euteiches* specific gene copies. Three technical PCR replicates were performed for each concentration.

As the classic methods using the DSI and the counting of oospores are time-consuming and tedious, especially with the large number of root samples, we developed primers and probes to evaluate the disease severity using qPCR. Figure 4 shows the relation between the *A. euteiches* specific gene and the qPCR threshold cycles. The efficiency of the qPCR was 99.58% with high sensitivity to detecting one copy of the *A. euteiches* specific gene (Figure 4).

**Figure 5:**
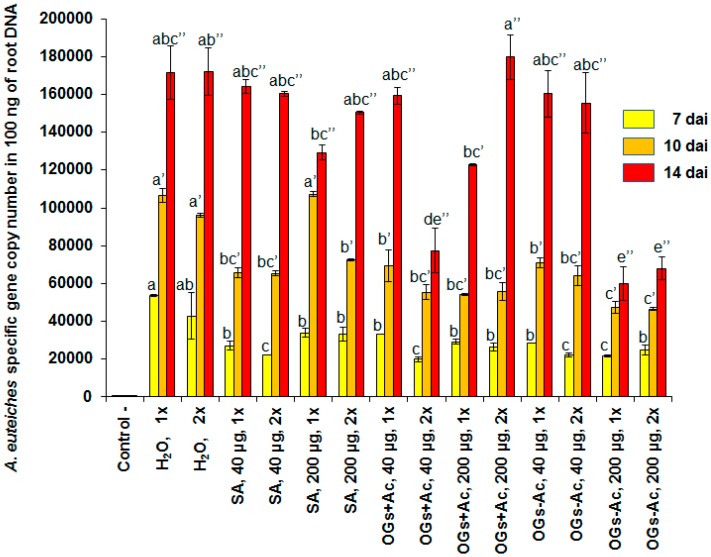
*Aphanomyces euteiches* specific gene copy numbers in 100 ng of the total DNA extracted from root samples collected at 7, 10 and 14 dai (days after inoculation). Pea plants injected in the upper two rachises with 20 µL/rachis of salicylic acid (SA), acetylated oligogalacturonides (OGs + Ac) or nonacetylated OGs (OGs − Ac). The elicitor injections were done once (1×) two weeks before inoculation (wbi) or twice (2×) two and one wbi. The final elicitor concentrations were 40, 80, 200 or 400 µg/plant. Controls were injected with water. The values shown are means with SD (*n* = 5). Different lower-case letters indicate significant differences between treatments according to the Tukey test (*p* ≤ 0.05).

The results in Figure 5 show the *A. euteiches* specific gene copy numbers in 100 ng of total DNA (AESG_100ng_) extracted from root samples collected at 7, 10 and 14 dai. Elicitor efficiency was calculated by comparing the levels of AESG_100ng_ in the inoculated plants after the elicitor pretreatment with those in the inoculated control plants without elicitor pretreatment. The well-known endogenous elicitor salicylic acid (SA) was used as a reference. No significant differences in AESG_100ng_ were observed between all the inoculated control modalities, injected or non-injected with water, at the three observation dates. These controls were grouped and used as repetitions for non-treated inoculated controls. Plants treated with the high doses (200 and 400 µg/plant) of SA showed phytotoxicity symptoms of brown necrotic lesions on the leaves and the injected rachises were dead. On the other hand, unstable protection efficiencies (50, 38 and 4%) and (48, 32 and 7%) were recorded in root samples collected at 7, 10 and 14 dai, respectively, as a response to the injection with SA (40 µg/plant) once at two wbi or twice (= 80 µg/plant) at two and one wbi (Figure 5). The same phytotoxicity symptoms, but less severe than those seen with SA, were observed in plants injected with the high dose (400 µg/plant) of OGs − Ac and OGs + Ac. Unstable protection was observed with the one-date injection modalities (two wbi) with 40 µg/plant of OGs − Ac (74, 33 and 6%) and OGs + Ac (38, 35 and 7%) at 7, 10 and 14 dai, respectively (Figure 5). However, significant and stable protection efficiencies of 58, 45 and 55% were recorded at 7, 10 and 14 dai, respectively, in the plants treated twice, at two and one wbi, with 40 µg/plant OGs + Ac at each date (=80 µg/plant) and of 59, 56 and 65% in the plants treated at only one date (two wbi) with 200 µg/plant OGs − Ac (=2.5 folds more than OGs + Ac) (Figure 5). The differences between these two treatments were not significant. The root samples of these two efficient treatments were used for gene expression studies.

### 2.2. Gene Expression

**Figure 6:**
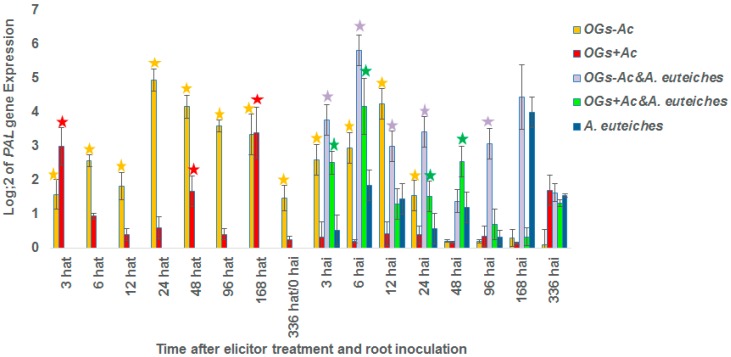
*PAL* gene expression ratio at 3, 6, 12, 24, 48, 96, 168 and 336 hours after treatment (hat) with water, 80 µg/plant of acetylated oligogalacturonides (OGs + Ac) or 200 µg/plant of nonacetylated OGs (OGs − Ac). The elicitor injections were done two weeks before inoculation (wbi) and two and one wbi for OGs − Ac and OGs + Ac, respectively. After inoculation with 10^5^ *Aphanomyces euteiches* zoospores/plant, *PAL* gene expression was followed over the same time course (hours after inoculation (hai)). The values shown are means of five repetitions. **☆** Stars indicate gene induction ≥2-folds and significant differences between elicitor treatments and inoculated non-treated control according to the Tukey test (*p* ≤ 0.05).

The expression of Pathogenesis Related protein 1 (*PR1*), *1*,*3 β glucanase* and phenylalanine ammonia-lyase (*PAL*) genes was followed at 3, 6, 12, 24, 48, 96, 168 and 336 hat and using the same time course after inoculation (hai) in root samples harvested from plants injected twice at two and one wbi with 80 µg/plant of OGs + Ac (40 µg/plant/date) or once at two wbi with OGs − Ac (200 µg/plant). No upregulation of the three tested genes was detected in the controls injected with water in comparison with the control non-injected and non-inoculated. Neither elicitor treatments nor inoculation with *Aphanomyces* showed any expression changes for the *PR1* and *1*,*3 β glucanase* genes over the tested time course. However, significant upregulation (≥2 folds) of the *PAL* gene was recorded at 6, 12, 48, 168 and 336 hai with *A. euteiches* (Figure 6). On the other hand, high and early induction (3 hat) of *PAL* was observed in roots pretreated with both OG compounds. This active response of *PAL* over the tested time course in non-inoculated plants was higher and more stable with OGs − Ac (until 360 hat) than with OGs + Ac (until 168 hat) (Figure 6). The highest recorded *PAL* expression value was found at 168 hat in the plant roots pretreated with OGs + Ac (12.9-fold) and 31-fold at 24 hat in the plant roots pretreated with OGs − Ac. At the inoculation time (336 hat), *PAL* expression in OGs − Ac samples was upregulated 2.8-fold and this value increased strongly after inoculation to reach 13.8, 56.7, 8.0, 10.8 and 8.4-fold at 3, 6, 12, 24, and 96 hai, respectively (Figure 6). These values were significantly higher than those in the inoculated non-treated control which were up-regulated 1.4, 3.6, 2.7, 1.5 and 1.3-fold, respectively. In the case of OGs + Ac, *PAL* expression was 0.3-fold at the time of inoculation and upregulated after inoculation to reach 5.7-, 18.2-, 2.9- and 5.9-fold at 3, 6, 24 and 48 hai, respectively (Figure 6). These values were significantly higher than those in the inoculated non-treated plants which were up-regulated 1.4, 3.6, 1.5 and 2.5-fold, respectively.

**Figure 7:**
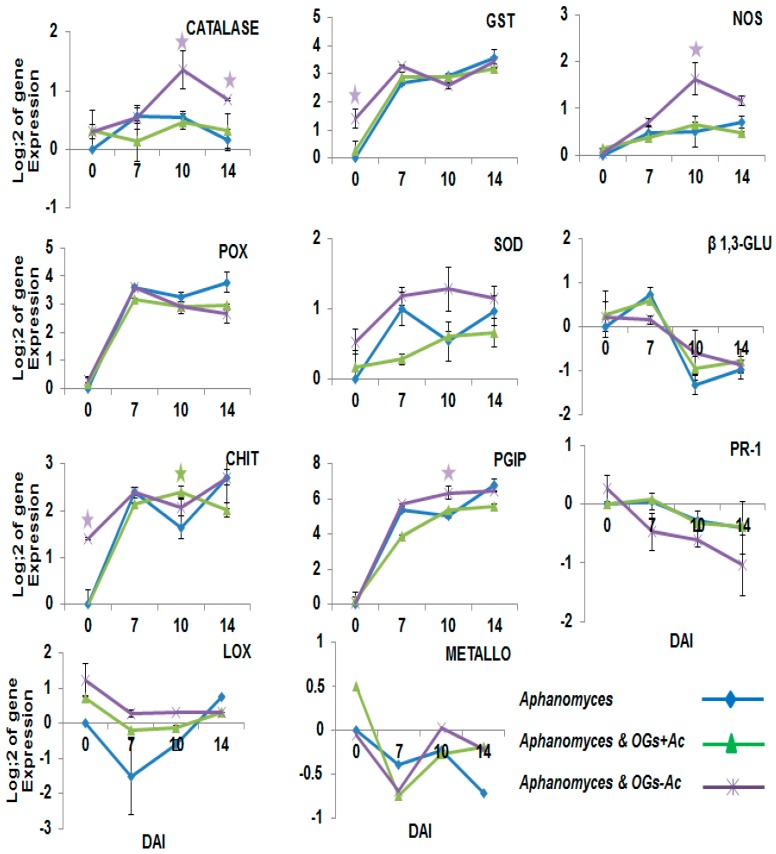
Pea gene expression ratio at 0 (just at the time of root inoculation), 7, 10 and 14 days after inoculation with 10^5^ *Aphanomyces euteiches* zoospores/plant. Pea plants were injected with acetylated oligogalacturonides (OGs + Ac) one and two weeks before inoculation (final concentration = 80 µg/plant) or with nonacetylated OGs (OGs − Ac) two (wbi) (final concentration = 200 µg/plant). Controls were injected with water. The values shown are means of 5 repetitions. **☆** Stars indicate gene induction ≥2-folds and significant differences between elicitor treatments and inoculated non-treated control according to the Tukey test (*p* ≤ 0.05).

**Figure 8:**
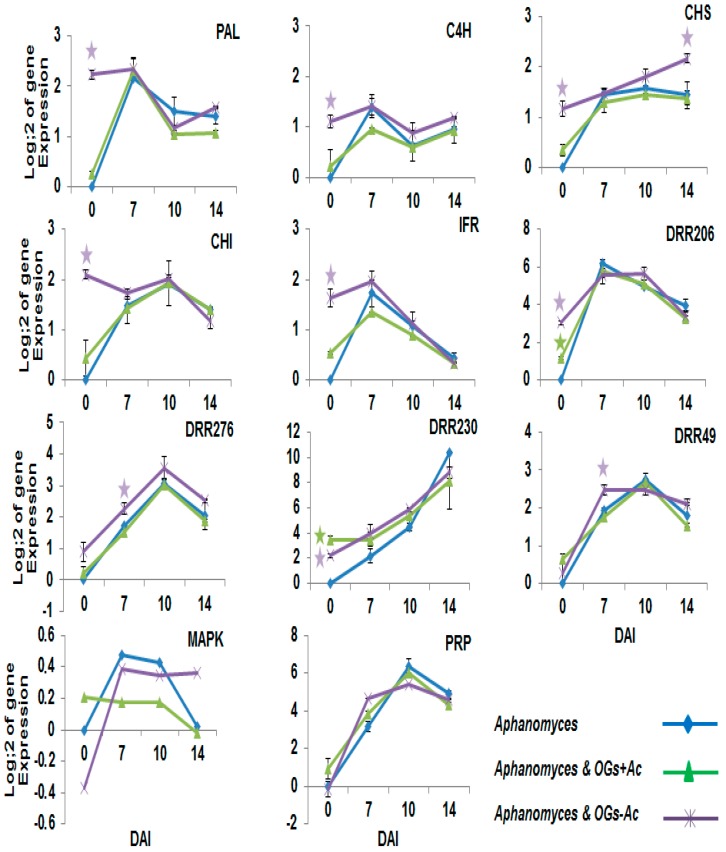
Pea gene expression ratio at 0 (at the time of root inoculation), 7, 10 and 14 days after inoculation with 10^5^ *Aphanomyces euteiches* zoospores/plant. Pea plants were injected with acetylated oligogalacturonides (OGs + Ac) one and two weeks before inoculation (final concentration = 80 µg/plant) or with nonacetylated OGs (OGs − Ac) two (wbi) (final concentration = 200 µg/plant). Controls were injected with water. The values shown are means of 5 repetitions. **☆** Stars indicate gene induction ≥2-folds and significant differences between elicitor treatments and inoculated non-treated control according to the Tukey test (*p* ≤ 0.05).

The expression changes in twenty-two pea defense genes as a response to OG pretreatments were followed using RT-PCR in root samples at 0 (just at the time of root inoculation), 7, 10 and 14 dai with *A. euteiches* in three modalities: inoculated without OG elicitor pretreatment; inoculated and pretreated with 80 µg/plant OGs + Ac; and inoculated and pretreated with 200 µg/plant OGs − Ac. These modalities were compared with the inoculated untreated controls. The results in Figure 7 show that at the inoculation time (0 dai = 14 dat), except for *GST* with OGs − Ac pretreatment, no significant induction of the genes involved in the ROS pathway (*SOD*, *POX*, *Catalase*, *NOS*, *Metallothionein*) was observed. After inoculation, only the *Catalase* and *NOS* genes were significantly upregulated at 10 dai in plants pretreated with OGs − Ac compared to their levels in the inoculated non-treated control. In addition, no significant induction was observed for the genes coding for *MAPK* and *PRP* (Figure 8). For the genes coding for pathogenesis-related proteins, *PR1*, *β1*,*3 glucanase*, *chitinase*, *DRR230*, *DRR276*, *DRR206* and *DRR49*, significant inductions of *chitinase* expression, at 0 and 10 dai, were recorded in the plant roots pretreated with OGs − Ac and OGs + Ac, respectively. At 7 dai, *DRR276* and *DRR49* were recorded in the plant roots pretreated with OGs − Ac (Figure 7 and Figure 8). The *DRR206* and *DRR230* genes showed significant upregulations at 0 dai and at 0 and 7 dai respectively, in the plants pretreated with the two OG compounds (Figure 8). However, no significant activation was observed for *PR1* and *β1*,*3 glucanase* genes with either of the two OG elicitors. The *PGIP* gene, coding for polygalacturinase inhibitor protein enzyme (marker gene for the basal defense), and *LOX*, coding for lipoxygenase enzyme (marker gene of the jasmonic acid pathway), showed no significant upregulation except for *PGIP* at 10 dai and only with OGs − Ac.

For the genes involved in the phenylpropanoid and phytoalexin pathways (*PAL*, cinnamate-4-hydroxylase (*C4H*), chalcone synthase (*CHS*), chalcone isomerase (*CHI*), and isoflavone reductase (*IFR*)), and known as SA gene markers, these were all significantly upregulated at the time just before inoculation (0 dai) in the plants pretreated with OGs − Ac. Only the *CHS* gene was significantly upregulated at 14 dai in roots from plants pretreated with OGs − Ac compared to those untreated and inoculated with *A. euteiches*. None of these genes showed upregulation at the time of inoculation in the plants pretreated with OGs + Ac.

## 3. Discussion

The oomycete *Aphanomyces euteiches* causes up to 80% crop loss in pea (*P. sativum*). To date, disease control measures are limited to crop rotation and no resistant pea lines or efficient fungicides are available. The present study aimed to investigate the potential of citrus-derived OGs to stimulate defense mechanisms in pea roots against *A. euteiches*. We tested two OGs fractions with DPs ranging from 2 to 25, nonacetylated (OGs − Ac) and acetylated at 30% (OGs + Ac).

Our results revealed no protection efficiency against *A. euteiches* root rot as a response to the commercial elicitors tested, Chitosan^®^ and Iodus^®^, as well as the heated inoculum of *A. euteiches* zoospores. In parallel, SA showed unstable protection efficiencies and phytotoxicity symptoms with the higher doses tested.

In contrast, plants injected with the OGs showed a significant disease reduction. High and stable protection efficiencies (>45%) were recorded in plants pretreated with OGs. On the other hand, the acetylation of OGs reduced the efficient dose more than two times compared to the nonacetylated OGs. It has been shown that the degree of OGs methylation clearly influences plant defense responses in wild strawberry against *B. cinerea* [13], in tomato against *Ralstonia solanacearum* [11], in wheat against *Puccinia graminis* f. sp. *tritici* [14] and against *B. graminis* f. sp. *tritici* [15]. Indeed, the ability of bacterial or fungal necrotrophs to produce pectin methylesterases (PME) is often related to a successful initiation of the infective process. Pectin is synthesized in a highly methylesterified form and is subsequently de-esterified in muro by PME. De-esterification makes pectin more susceptible to the degradation by pectic enzymes such as endopolygalacturonases and pectate lyases [28]. Wayra and Bari [11] observed in their immunohistochemical studies constitutive differences between tomato genotypes susceptibility to *R. solanacearum* which manifested in methyl-ester distribution of homogalacturonan (HG), arabinan and galactan side chain composition of rhamnogalacturonan I (RG I) and arabinogalactan-protein (AGP) in the xylem parenchyma and in vessel cell walls. They suggested that *R. solanacearum* PME may act on HGs of the susceptible plant in a non-blockwise deesterification pattern, while in the resistant genotype the constitutive, more blockwise methyl-ester distribution and the increased AGP content and higher side chain branching of RG I in vessel cell walls may inhibit easy degradation. However, it is not yet clear how esterification affects OGs biological activity [29].

Gene expression analysis showed no upregulation of the genes involved in the ROS pathway as a response to OGs except for the *GST* gene with OGs − Ac. However, a priming effect in the expression of the *catalase* and *NOS* genes were observed after challenging the OGs-treated plants with *A. eutiches*. Most interestingly, it is known that elicitors such as jasmonic acid, salicylic acid, nitric oxide and superoxides or their precursors do not significantly enhance the resistance induction of pea, and at high concentrations can negatively affect resistance [30]. The induction of the *GST* gene could be explained by its broad spectrum of functions in plants, such as transport and storage of reduced sulfur, detoxification, and antioxidation, as well as a role as cofactor in enzymatic processes, protein reduction, and in phytochelatin by complex-binding heavy metals that have thiol affinity [31,32]. The observed priming induction of the *PGIP* as a response to OGs − Ac indicates the importance of this gene against *A. euteiches*. PGIP are plant extracellular leucine-rich repeat proteins that effectively and specifically bind and inhibit fungal [33] and bacterial [19,20] polygalacturonases and inhibit further invasion of these pathogens. The down-regulation of *PGIP* at early time points after inoculation could be attributed to virulence factors released by the pathogen to suppress the host resistance and facilitate host colonization [34].

Looking at the genes coding for pathogenesis-related proteins, only *DRR276* and *DDR49* genes were upregulated as a response to OGs − Ac. Both the *PR10 DRR49*, *DRR276* pea genes code for a protein homologous with RNase [35,36]. Transgenic ‘Shepody’ potatoes possessing the pea gene DRR49 displayed resistance to potato early dying disease (*Verticillium dahliae*) [37]. However, the utility of this gene may be related to the close homology of its product to other plant allergens [38].

In parallel, the *chitinase*, *DDR206* and *DDR230* genes were significantly overexpressed as a response to both OGs. The stimulation of *chitinase*, which codes for an enzyme that digests chitin in the fungus cell wall, could be an important mechanism against *A. euteiches*, which contains 10% of chitin in its wall structure [39]. It has been reported that the pea defense gene *DRR206* confers resistance to black leg (*Leptosphaeria maculans*) disease in transgenic canola (*Brassica napus*) by inhibiting fungal germination and decreasing hyphal growth at inoculation sites [40]. Recently, Seneviratne et al. [41] investigated *in planta* the biochemical function of the DRR206 and reported that the metabolite associated with its gene induction is the pinoresinol monoglucoside. The pinoresinol is a member of a large, structurally diverse, class of lignans, which have a wide range of physiological and pharmacologically important properties [42,43]. Because of their pronounced biological (antimicrobial, antifungal, antiviral, antioxidant and anti-feedant) properties, a major role of lignans in vascular plants is to apparently help confer resistance against various opportunistic pathogens and predators [41]. The *DDR230* gene that codes for proteins with a high cysteine content, called defensins [44], has been found previously actively expressed in the pea endocarp during the resistance response to *Fusarium solani* f. sp. *phaseoli* [45]. Defensins are antifungal products of some PR genes and one of the first defensin genes cloned, *DRR230*, was isolated from pea [44]. When it was overexpressed in canola, extracts of these plants inhibited the in vitro germination of *L. maculans* [40]. Pea defensins have also been used in the biological control of blue mold in apple [46]. The mode of action of pea defensins corresponds to that of similar highly-conserved antimicrobial peptides present in a broad range of biological organisms [47] and could consist of inducing the membrane destabilization/permeabilization required for fungal growth, inhibiting protein synthesis, enzyme activity, and ion channels [48].

The earlier induction of the *PAL* gene, marker of the phenylpropanoid and phytoalexin pathway, in pea roots infected with *A. euteiches* indicates the importance of this pathway in pea defense reactions. The preventive induction of this pathway has previously been reported as one of the important strategies to control root legume diseases [49]. Before inoculation with *A. euteiches*, a high and early induction (3 hat) of *PAL* was observed in pea roots as a response to both OGs elicitors, with higher levels with OGs − Ac. After root inoculation with *A. euteiches*, the induction of *PAL* as a response to both OGs was primed to be significantly higher than its levels in inoculated non-treated plants. This priming effect was observed during the first 4 dai. In fact, PAL enzyme catalyzes the first step in the phenylpropanoid pathway toward the biosynthesis of a large variety of products, including antimicrobial phytoalexin compounds such as pisatins, antioxidant protectants such as flavonoid compounds, and precursors of lignin [50,51,52]. Pisatin production is dependent on PAL and a series of other secondary enzymes, such as TCAH, CHS, CHI, and IFR [49,53]. At the inoculation time, all of these genes were significantly upregulated as a response to OGs − Ac pretreatment. Previous investigations reported that multiple genes control the pathogenicity of fungal isolates on pea and of these, the gene for pisatin demethylase enzyme (PDA) [54], which detoxifies the phytoalexin pisatin [55], is considered the most important. It has also been reported that all isolates without the *PDA* gene were essentially non-pathogenic on peas [30,56] showing the importance of the pisatin pathway in the pea defense against pathogen attack. Interestingly, both pinoresinol monoglucoside and pisatin were found co-localized in pea pod endocarp epidermal cells and associated with *CHS* and *DRR206* gene expression, indicating that both pisatin and pinoresinol monoglucoside function in the overall phytoalexin responses [41].

In conclusion, acetylated and nonacetylated oligogalacturonides confer protection in pea against *A. euteiches* root rot. Acetylation allows a significant reduction of the efficient elicitor dose of OGs, suggesting that acetylation is necessary for some specific responses. The induction of the antifungal defensins, lignans and the phytoalexin pisatin pathways and their priming effect in the expression of the basal defense, SA and ROS gene markers could explain their stable and synergetic protection efficiency. Taken together, acetylated OGs are interesting elicitors to stimulate defense mechanisms in pea. 

## 4. Materials and Methods

### 4.1. Elicitor Compounds 

OGs were produced at the Laboratoire des Polysaccharides Microbiens et Végétaux (Université Jules Verne, Amiens, France). A mixture of OGs was obtained by thermal degradation of polygalacturonic acid from citrus fruit following the same methods described previously in Randoux et al. (2010)*.* OGs with polymerization degrees (DPs) of 2 to 25 were selected by sequences of purification using acetic acid and isopropanol and then mixed together. This mixture of OGs is hereafter referred to as the nonacetylated OGs (OGs − Ac). Dried OGs − Ac were acetylated using acetic anhydride. After addition of H_2_O, the preparation was dialyzed and the acetylated galacturonides were freeze-dried. Samples were then dissolved in D_2_O, 99.96% D; the final concentration was 15 g L^−1^. The degree of acetylation was calculated by integration of the signals in the downfield, upfield, and acetyl regions as described in the literature [57] and OGs with a degree of substitution (DS) of 30% (OGs + Ac) were used in further experiments. OGs + Ac were characterized by the presence of acetyl groups linked on either the C2 or C3 of galacturonan residues, as described in [15]. For all the esterification procedures applied, the DS and the distribution of acetyl groups on the galacturonan residue were always the same.

### 4.2. Plant Material and Growth Conditions

*Pisum sativum* L. commercial cv. Alezan, highly susceptible to pea root rot caused by *Aphanomyces euteiches*, was used in the experiments. The seeds were sterilized by immersing them in 70% ethanol alcohol for 5 min and then in a solution of 1% NaOCl for 15 min with three intervals of washing in sterilized distilled water. Plants were grown from seeds in 0.52 L pots (9 × 9 × 8 cm) filled with autoclaved vermiculite in a growth chamber at 20 ± 2 °C and under a photoperiod of 16 h daylight with a light intensity of 150 μmol m^−2^ s^−1^ photon flux density supplied by high-output white fluorescent tubes (Philips Master Cool White 80 W//865, Lamotte Beuvron, France). Plants were irrigated daily and once a week with 25% Murashige and Skoog nutritive solution (Murashige and Skoog medium, Sigma-Aldrich, Saint Louis, Mo, USA).

### 4.3. Inoculum Preparation and Inoculation

*A. euteiches* was cultured on corn meal agar medium (Sigma-Aldrich, St. Quentin Fallavier, France) at 18 °C in the dark for three days. Then, zoospores of *A. euteiches* were produced in a mineral salt solution as described by Carman and Lockwood [58]. At 5 weeks post-germination, 25 mL of water containing 10^5^ zoospores was added to each pot on top of the vermiculite. Control plants were irrigated with the same amount of water without zoospores. Three, seven, ten and fourteen days after inoculation, plant roots were harvested and colored with lactophenol cotton blue stain for microscopic observations, or conserved at −80 °C until DNA extraction to follow the disease progression using qPCR. *P. sativum*-*A. euteiches* compatibility was evaluated at 7, 10 and 14 dai using the DSI ratings from 1 to 5 as follows: 1 = no necrosis of roots and hypocotyls; 2 = slight necrosis of roots and hypocotyls; 3 = necrosis of roots and lower hypocotyls, slight chlorosis of cotyledons, and moderate stunting of stem; 4 = extensive necrosis of roots, hypocotyls, cotyledons, and severe stunting of stem; 5 = plant death. In the same samples, the percentage of roots containing more than 50 oospores was determined in samples of one hundred 1-cm root fragments per condition.

### 4.4. Protection Assay

All tested elicitors were dissolved in water. OGs − Ac and OGs + Ac elicitor solutions were prepared at 1 and 5 g L^−1^. Solutions of SA (Sigma-Aldrich, St. Quentin Fallavier, France), Chitosan^®^ (Sigma-Aldrich, St. Quentin Fallavier, France), Iodus^®^ (Goëmare, Saint Malo, France) at 1 g L^−1^ were tested. An inoculum of *A. euteiches* (AE) zoospores (10^5^ zoospores mL^−1^), heated at 100 °C for 10 min to kill the zoospores, was also tested for its elicitor activity. Plants were injected with each elicitor compound or with water for the control plants on the upper one or two proximal rachises (20 µL/rachis). Different elicitor doses were used: 20, 40, 80, 200 and 400 µg/plant. Depending on the date of elicitor injection, three timing modalities were carried out: at 3-weeks, at 4-weeks, or twice at 3- and 4-weeks post-germination. The plants were then inoculated at 5-weeks-old as mentioned above. Three plant control modalities were used: injected with water and inoculated; non-injected and inoculated; non-injected and non-inoculated. The roots from controls and treated plants were harvested at 7, 10 and 14 dai for disease observation and at 3, 6, 12, 24, 48, 96, 168 and 336 hours after treatment (hat) and at the same hours after inoculation (hai) for gene expression studies.

### 4.5. DNA and RNA Extraction 

For *A. euteiches* DNA quantification and gene expression experiments, roots were harvested and stored immediately in liquid nitrogen and subsequently used for DNA and RNA extraction. Total DNA and RNA were isolated from *P. sativum* roots with the DNeasy and RNeasy plant mini kits (Qiagen, Les Ulis, France), respectively, in accordance with the manufacturer’s recommendations. DNA and RNA concentrations and qualities were evaluated using absorption values at 260 and 280 nm, and RNA quality was also checked by gel electrophoresis.

### 4.6. Real-Time PCR 

#### 4.6.1. Real-Time Quantitative PCR (qPCR)

To quantify infection levels of *A. euteiches*, primers and TaqMan minor groove binder probes (Table 1) were designed, using the Primer Express 3 software (Applied Biosystems, Foster, CA, USA), to target a 61-bp fragment of the *A. euteiches* specific gene (GenBank accession No. AF228037.1 [59]). A TaqMan assay was carried out in 25 μL of a reaction mixture containing the following: 12.5 μL of universal TaqMan PCR Master Mix (Applied Biosystems, Foster, CA, USA), 0.3 μM of each primer, 0.2 μM of probe, 200 ng of DNA and water up to a volume of 25 μL. The conditions of qPCR amplification were the following: 10 min at 95 °C, followed by 40 cycles of 15 seconds at 95 °C and 1 min at 60 °C. qPCR analysis of the *A. euteiches* specific gene was calibrated from 10^2^ to 10^7^ copies by serial dilution of the appropriate cloned target sequence.

#### 4.6.2. Real-Time Reverse Transcription PCR (RT-PCR)

The cDNAs were prepared as follows; 1 µg of total RNA was added to 1.5 µg of oligo(dT)_15_–dNTP (2.5 mM each) and made up to a final volume of 11.5 µL with sterile distilled water. RNA was denatured for 5 min at 70 °C and placed on ice, and then 5 µL of Moloney murine leukemia virus (MMLV) 5× reaction buffer, 300 U of MMLV reverse transcriptase, and 80 U of RNase inhibitor were added. First-strand cDNA was synthesized at 25 °C for 15 min, followed by incubation for 50 min at 42 °C and 2 min at 96 °C. Then, gene-specific fragments were amplified by real-time PCR using the defense gene specific primers and probes listed in Table 1, which were designed using the Primer Express 3 software. The TaqMan assays were carried out as mentioned above. All PCR experiments were carried out using an ABI PRISM 7300 sequence detection system (Applied Biosystems, Foster, CA, USA). The *Mtgap1* gene was used as an internal reference control for equivalent reverse transcription to cDNA and equivalent amplification in the PCR. Expression ratio for each cDNA was calculated relatively to corresponding controls, injected with water, using the 2^−ΔΔCt^ method as described by Livak et al., 2001 [60].

### 4.7. Statistical Analyses 

Five technical repetitions were used for each experimental condition and four separate experiments were carried out. For all experiments, significant differences were evaluated using the Tukey test at *p* ≤ 0.05.

## Figures and Tables

**Table 1:** Oligonucleotide primer sequences of pea defense genes.

Gene Name	GenBank	Forward, Reverse Primers and Probes (5′–3′)	*T*_m_ (°C)	Amplicon Length
	Accession N°			
*Aphanomyces euteiches*	AF228037	TTTTGGAACACCCAAACGTACTG	58	61
		AGTCCAAGAGGCATTCGACAA	58	
		ACGCTGAGCTTGAC	68	
**Housekeeping genes**				
GAPDH (Mtgap1) ^1^	X73150	GTCTTTGCACACAGGAACCCA	59	123
		GGCACCACCCTTCAAATGAG	59	
		CCCATGGGCCAGCAC	70	
**Defense and cell rescue**				
Pathogenesis protein 1 (PR1)	AJ586324	CCTTCCCCTCATGGCTATCC	59	69
		TGTGGTGAGTTTTGAGCATATGAGA	59	
		AGTACTATCCACATCAACAC	68	
Proline-rich protein (PRP)	AJ233399	TGGCTTCCTTAACCTTCCTACTGT	58	64
		TTGGCAAACCCTTGAGGAAT	58	
		ACTCCTTCTTGCTCTTAT	68	
Mitogen-activated protein kinase (MAPK)	X70703	CATTCCGCGAATGTTTTGC	58	59
		TTGGCGTTCAGGAGAAGGTT	58	
		AGGGACTTAAAACCC	70	
**Reactive oxygen species (ROS) **				
Superoxide dismutase (SOD)	AB087845	CCATCATAGGAAGGGCTGTTGT	59	63
		CGTGACCACCTTTCCCAAGA	59	
		CCATGCCGATCCTGAT	70	
Peroxidase (POX)	AB193816	ATGCAAGAACAGCAAGCCAAA	59	69
		GGGTTGCAAGGTCAGATGATG	59	
		AACAGTCAAATCCC	70	
Nitric oxide synthase (NOS)	AY672712	GGCGGTGGTCAGGGTCTT	59	63
		CCCTTTGGGACACGCTTTT	59	
		TGGAAAGAATGGATCTATT	68	
Glutathione S-transferase (GST)	AB087837	GAGAATGCCCTTGGTAAATTTGA	58	70
		ACGCAATATCCACCAAACTGAAT	58	
		CCCCTTCCTTCTTGGTC	69	
Catalase (Cat)	X60169	CCAAGTGGTCTCACCACAACAAT	59	69
		TGACCTCCTCATCCCTGTGAA	59	
		CCATGAGGGTTTCATG	69	
Metallothionein (Metalo)	AB176564	TCCGGCGAAGATCCAGTTT	59	69
		CCACACTTGCAGCCACCAT	59	
		TGGTGCTGAAATGAGTG	69	
**Cell wall proteins & Basal defense**				
Chitinase (Chit)	L37876	CCTTCAAGACCGCTTTATGGTT	58	64
		ACGTCGTGGCAGGATGGTT	60	
		ACGCCTCAGTCACCT	68	
Beta-1,3-glucanase (β 1,3-Glu)	S51479	TGGAATTGGTTGGGTGAATGT	58	65
		TTGCAGAGCCTCCATCTGAA	58	
		TTGTTTCTGAGAGTGGTTG	68	
Polygalacturonase inhibiting protein (PGIP)	AB087839	CAGTGCTTTTCGGGAGCAA	59	66
		CAAACGACAGCAAGTTCCTTGA	59	
		AAAGGACACAGATACTTGAT	69	
**JA signaling pathways **				
Lipoxygenase (LOX)	X17061	TGATCCGCGGTCTTCAAGAG	59	60
		CACCGTATTCTGCGGGATCT	59	
		TTCCTCCGAAAAGC	69	
**Phenylpropanoid & Phytoalexin pathway**				
Phenylalanine ammonia lyase (PAL)	D10001	GCACTTAGAACTTCACCGCAATG	60	
		GAAAGTTTCCACCATGCAAAGC	60	
		CCCTTTGATTGATGTTTC	69	
Cinnamate-4-hydroxylase (C4H)	U29243	GCCATAACCGCCATCACAAT	59	61
		GGGCCAGGAGGGAGTTTGAA	59	
		AACTCCGCGGCAAA	68	
Chalcone isomerase (CHI)	U03433	GCTGCAGCATCCTCCATCA	58	56
		CACCGCTGGGAACTCATGT	58	
		CGCAATCCACGTCGAG	67	
Chalcone synthase (CHS)	D10662	GACATGGTGGTCGTCGAGGTA	58	70
		GCCCCCATTCTTTTATAGCTTTC	58	
		AGACTAGGGAAAGAGGCT	70	
Isoflavone reductase (CHR)	S72472	CTTTTGGCGTTGTACCATTCG	59	68
		TCTTTGGCAGGGTCAATCTCA	59	
		AACAAATAAAGGGAGATGCAG	70	
**Disease resistance response (DRR)**				
DRR230	AJ308155	TTGCAGGAACAACGAGCACTT	60	61
		GCACCAGCAGCGAAAATCAT	60	
		CTCAGTGGGAGGTGCA	69	
DRR276	M18249	TGCTGACACTCTTACTCCAAAGGT	58	66
		CCGTTTCCTTCAACAATTTCG	58	
		TTGATGCCATCAAAAGTA	69	
**Disease resistance response protein (DRR)**				
DRR49	X13383	GGTGATGCTGCTCCTAGTGAAGA	58	66
		CTTGAAAAGACCATCCCCCTTA	58	
		CAACTCAAGACTGACAAAG	68	
DRR206	M18250	GCTGGAGCTGACCCAATTGT	59	68
		AAGAAATCTCCAGTACCACCAGTGA	59	
		CCAAAACTAGAGATATTTCT	69	

^1^ The GAPDH (glyceraldehyde-3-phosphate dehydrogenase) genes were used as an internal reference control for equivalent amplification in the PCR.
